# The effect of ovariectomy on production and physiological parameters and the physicochemical quality of meat in Rhode Island Red hens

**DOI:** 10.1016/j.psj.2026.107983

**Published:** 2026-10-17

**Authors:** Joanna Obrzut, Jolanta Calik

**Affiliations:** National Research Institute of Animal Production, Department of Poultry Breeding, 32-083 Balice k, Krakowa, Poland

**Keywords:** Poulards, Meat quality, Native breeds, Sensorial evoluation

## Abstract

The aim of the study was to evaluate the effect of ovariectomy on production and physiological parameters and the physicochemical quality of meat in Rhode Island Red (RIR) hens (K-22). The experimental material consisted of 80 hens. The day-old chicks were weighed, wing tagged, and then divided into two groups of 40 birds each (5 replicates of 8 birds each). The control group consisted of unsterilized hens (group I), while the experimental group (group II) consisted of hens that underwent ovariectomy at 10 weeks of age. The birds were fed *ad libitum* with the same feed mixtures and housed on litter under optimal environmental conditions at a stocking density of 5 birds/m². At the end of the fattening period, i.e., at 24 weeks of age, 10 birds from each group whose body weight was close to the group average were selected for slaughter. After slaughter, the birds were analyzed for production and physiological indicators. The physicochemical and sensory quality of the meat from hens and poulards was also evaluated.

The results showed that poulards achieve higher body weight and slaughter yield. In addition, they are characterized by a higher proportion of breast and leg muscles in the carcass, as well as higher fat content. Both the breast and leg muscles of the poulards exhibited better physicochemical parameters and higher sensory ratings. The research results confirm that RIR hens of the K-22 line are a valuable resource for poulard meat production. It can therefore be concluded that the ovariectomy of hens from conservation breeds is a valuable tool for creating a premium product that combines the preservation of genetic resources with the development of a high-quality market offering.

## Introduction

Poultry meat is currently one of the world’s main sources of animal protein, and forecasts indicate continued rapid growth in its production over the coming decade (OECD/FAO, 2023). Along with the growing scale of production and improving living standards among consumers, there is a clearly increasing interest in niche products characterized by higher quality and specific sensory attributes. In this context, native breeds are becoming increasingly important, particularly multipurpose breeds such as the Rhode Island Red (RIR), which – despite a slower growth rate compared to commercial broilers – provide meat with valuable sensory properties (Sokołowicz et al., 2016; Obrzut et al., 2021; Calik and Obrzut, 2023b; Yang et al., 2024).

According to Commission Implementing Regulation (EU), 2020/464, a poulard is a female *Gallus gallus* chicken intended for meat production, slaughtered at a minimum age of 120 days, which distinguishes it from both broilers and typical laying hens. For centuries, the poulard has been considered a luxury product in European cuisine, prized for its exceptional delicacy, tenderness, and juiciness (Krawczyk et al., 2018; Obrzut et al., 2018). Historical sources indicate that fattening poultry with high-quality feed, combined with ovariectomy, results in white, tender meat with excellent flavor (Calik, 2014). As late as the first half of the 20th century, poulards were an important element of court and rural cuisine and served as “a means of payment”, which underscores their economic and cultural significance. Today, the tradition of raising poulards continues, as exemplified by the “Mazovian Poulard”, which is listed among Poland’s traditional products, highlighting its characteristic conformation, distinct fat cover, and unique sensory qualities (https://www.gov.pl/web/rolnictwo/pularda-mazowiecka).

The procedure of surgical castration of poultry (caponization) is well known in rooster rearing, where it results in capons – birds with increased body weight, higher slaughter yield, greater fat content, and significantly better sensory characteristics of the meat compared to uncastrated individuals (Adamski et al., 2016; Calik et al., 2020; Calik and Obrzut, 2023a). Numerous studies have shown that in capons of native or locally adapted breeds, the breast and leg muscles are characterized by a lighter and more yellow color, better tenderness, higher juiciness, and a better sensory ratings compared to uncastrated males (Amorim et al., 2016; Calik et al., 2017). Similar effects – i.e., higher carcass weight, increased proportion of breast and leg muscles, changes in color parameters, and improved sensory quality – have also been demonstrated in poulards (Cui et al., 2016a,b; Obrzut et al., 2018; Krawczyk et al., 2018; Zhang et al., 2025). From a practical standpoint, caponization and poulardization are therefore viewed as a way to produce higher-quality meat intended for the premium market segment. Although there is a relatively extensive body of literature on the impact of castration on the meat quality of capons, much less attention has been devoted to poulards, especially those from native chicken breeds included in genetic resource conservation programs. For this reason, it is appropriate to conduct research on the impact of ovariectomy of hens from conservation breeds on meat quality, which will not only provide a better understanding of the physiological and technological consequences of poulard production but also assess the potential of these populations to produce a high-quality traditional product.

The objective of this study was to evaluate the effect of ovariectomy on Rhode Island Red (K-22) hens in terms of production and physiological indicators, as well as the physicochemical quality of the meat.

## Materials and methods

### Experimental design

The experimental subjects consisted of 80 Rhode Island Red (RIR) hens from line K-22. The day-old chicks were weighed, marked with wing tags, and then divided into two groups of 40 birds each (5 replicates of 8 birds each). The control group consisted of unsterilized hens (group I), while the experimental group (group II) consisted of hens that underwent ovariectomy at 10 weeks of age.

The stocking density in the poultry house during the experiment was 5 birds per square meter of usable floor space, and the environment in which the birds were kept was enriched with additional elements, such as perches, extra bales of straw, a sand bath, and a claw-trimming area. The average temperature during the first days of rearing was approximately 32°C; it was then gradually lowered as the birds aged, so that by day 35, it ranged from 18 to 20°C at a relative humidity of 65 %. The lighting program used consisted of 20-24 h of light per day until day 14, and 12-16 h of light per day starting on day 15. Zootechnical preventive measures included vaccinations against Marek’s disease, Newcastle disease, infectious bronchitis, Gumboro disease, and coccidiosis.

All birds were fed *ad libitum* using a three-phase feeding system. Feed formulations were developed at the National Research Institute of Animal Production for the purposes of this experiment. The composition of the components and the results of chemical analysis, in accordance with the AOAC (2000) methodology, are presented in Table 1.

**Table 1:** Composition and nutrient content of the diets used in the experiment (kg/100 kg).

Component	Phase I day 1 to week 7	Phase II weeks 8 to 16	Phase III weeks 17 to 20
Ground maize	41.35	40.45	35.70
Ground wheat	25.00	22.00	29.00
Ground triticale	-	5.00	7.00
Ground barley	-	5.00	7.00
Soybean meal	30.00	24.00	18.00
Ground limestone	1.25	1.30	1.20
Dicalcium phosphate	1.60	1.45	1.30
NaCl	0.30	0.30	0.30
Vitamin-mineral premix DKA-F (finisher) (0.5 %). kg	0.50	0.50	0.50
Crude protein (g)	204	184	165
Metabolizable energy (MJ) (kcal)	11.92 2850	12.05 2880	12.18 2910
Lys (g)	10.3	8.90	7.50
Met (g)	3.10	2.85	2.60
Ca (g)	8.95	8.60	7.90
P available (g)	4.10	3.80	3.50

### Ovariectomy procedure

Surgical ovariectomy was performed on Group II hens at 10 weeks of age (average live body weight: ∼1.0 kg) by a qualified veterinarian. Local anesthesia was induced via infiltration of 2 % lidocaine hydrochloride at the surgical site. A modified lateral laparotomy approach was used. Feathers over the left flank were plucked, and the skin was thoroughly disinfected with povidone-iodine. A 2–3 cm longitudinal skin incision was made between the last two ribs on the left lateral flank. The abdominal muscle layers and peritoneum were carefully incised to access the abdominal cavity. The left ovary was exposed using sterile retractors, blunt-dissected from its attachment at the ovarian pedicle, securely ligated with sterile suture, and excised completely. The surgical site was inspected for complete tissue removal and hemostasis. The muscle and skin layers were sutured, and topical antiseptic and antibiotic sprays were applied to prevent postoperative infection.

### Ethics statement

The procedures for this experiment complied with the requirements of the 1st Local Ethics Committee for Animal Experiments in Kraków (Poland), as set forth in Resolution No. 514/2021 dated April 28, 2021. In accordance with European Union law, Directive, 2010/63/EU, enforced in Poland by Legislative Decree No. 266/2015 of the European Parliament and of the Council on the protection of animals used for scientific purposes, applied to this study.

### Collection of samples

At the end of the fattening period, i.e., at 24 weeks of age, 10 birds (2 birds from each of the 5 replicates) with the average body weight in the group were selected from each group for slaughter. An additional criterion for selecting poulards was a comb that was significantly smaller than that of hens, which occurred in 100 % of the birds. The pullets and poulards were not fed for 12 h prior to slaughter, but were provided with constant access to water. Euthanasia was performed by a trained person using the decapitation method, following prior electrical stunning with a KOMA STZ 6 stunner (KOMA, Świdnica, Poland).

### Analytical methods

#### Biochemical blood analyses

During the rearing period, blood was collected from 8 birds in each group to determine blood biochemical parameters. Blood was collected from the wing vein into lithium heparin tubes (MPW-52, MPW Med. Instruments) and centrifuged; the plasma separated by centrifugation was transferred with a pipette to Eppendorf tubes. At 10, 12, and 24 weeks of age, estradiol concentrations were determined using Snibe reagents with a Maglumi 600 chemiluminescence immunoassay analyzer (Middlesex London, United Kingdom). In addition, at week 24, the following plasma concentrations were measured: cholesterol (Chol), alanine aminotransferase (ALT), aspartate aminotransferase (AST), urea (UREA), creatinine (Crea), triglycerides (TG), amylase (AMYL), and lipase (LIPA). Biochemical parameter levels were analyzed using a Mindray BS-120 biochemical analyzer with Spinreact reagent kits (Girona, Spain).

#### Physical analysis of meat

After the birds were euthanized and bled out, standard post-slaughter processing was performed (scalding, plucking, and evisceration). The effectiveness of the procedure was also verified (absence of ovaries). The chilled carcasses were subjected to a slaughter analysis according to the method of Guo et al. (2017). The following percentages were determined: slaughter yield with giblets, slaughter yield without giblets, the proportion of breast and leg muscles, giblets (liver, gizzard, heart), bones, and abdominal fat.

• pH measurement: The pH of the breast muscles (*m. pectoralis major*) and thigh muscles (*m. biceps femoris*) was measured 15 min after slaughter (pH15) and after 24 h of carcass cooling (pH24). A Cyber Scan 110 pH meter equipped with a Hamilton glass blade-spear electrode specifically designed for direct muscle tissue insertion, electrode, calibrated in solutions with pH 4.0 and 7.0, was used for this purpose.
• Color measurement: The color of the carcasses and muscles was determined in the CIE L*a*b* system (lightness L*, redness a*, yellowness b*) using a Minolta CR 410 reflectance spectrophotometer (D65 light source, 2° observer). The result for the carcass was the average of five measurements (back, chest, legs), while for the muscles it was the average of two measurements taken on their inner surface immediately after filleting.
• Water holding capacity (WHC): determined using the method of Grau and Hamm (1953). The amount of juice mechanically squeezed from a meat sample onto filter paper (Whatman 1) was measured using a planimeter (e.g., Haff Digital Polar Planimeter No. 301).
• Natural drip loss and cooking loss: Meat juice loss (drip loss) was determined after 24 h of storing 80-gram samples at +4°C in airtight containers. Cooking loss was calculated after cooking the samples (80 g) in plastic bags in a water bath (100°C) until the core temperature of the muscle reached 76–78°C (cooking time: 14 min for breast, 16 min for thighs).
• Shear force: Meat tenderness was measured using a TA.XT.plus texture analyzer (Stable Micro Systems). Cylindrical samples with a diameter of 10 mm and a height of 30 mm were cut from the cooked muscles (which reached an internal temperature of 85°C). The samples were cut with a Warner-Bratzler knife (speed 2 mm/s) perpendicular to the muscle fiber orientation, and the final result was the average of three measurements.

#### Chemical analyses of meat

Chemical analyses were performed at the Central Laboratory of the National Research Institute of Animal Production in Kraków. The basic composition of muscle tissue (dry matter, total protein by the Kjeldahl method, crude fat by the Soxhlet method) was determined in accordance with AOAC (2000) methods. Cholesterol content was determined by gas chromatography. The fatty acid profile was analyzed using a Varian 3400 CX chromatograph on an Rtx 2330 column (105 m), following lipid extraction by the Folch method and BF_3_/methanol methylation. The following indices were calculated based on the proportions of individual acids and their groups: PI peroxidizability index (Arakawa and Sagai, 1986), TI thrombogenic index; AI atherogenicity index (Ulbricht and Southgate, 1991).

#### Sensory analysis of meat

Sensory analysis was conducted in accordance with the methodology of Baryłko-Pikielna and Matuszewska (2009). The evaluation was performed by a panel of 10 individuals – staff members of the National Research Institute of Animal Production – who were experienced in sensory evaluation techniques and had been familiarized with the meat evaluation methodology during preliminary sessions. The analysis took place in a sensory evaluation laboratory equipped with individual workstations. Throughout the testing, the laboratory environment was maintained at a temperature of 21°C (±0.5°C) and a relative humidity of 65 % using an air-conditioning system. White light lamps with a spectrum approximating daylight and an intensity of approximately 500 lx were used for illumination. Samples of breast and leg muscles were cooked without salt or spices to an internal temperature of 80°C and cut into pieces (1 × 2 cm or 15 g). These samples were then individually coded and presented to the panelists in a random order. Four attributes were assessed – namely aroma, juiciness, tenderness, and taste – using a scale ranging from 1 (lowest score) to 5 (highest score).

### Statistical analysis

Prior to testing, the Shapiro-Wilk test was used to assess the normality of the distribution. The test confirmed that the analyzed data conformed to a normal distribution. Statistical calculations were performed using Statistica 13.3 PL software (StatSoft Inc., Tulsa, OK, USA), and the results are presented as means ± SD (standard deviation). The data were analyzed using the *t*-test for independent samples. Results were considered statistically significant at the P ≤ 0.05 and P ≤ 0.01 levels.

## Results

Throughout the experiment, bird mortality was very low (Table 2). Only in group II was a mortality rate of 2.5 % (1 bird) observed, which was related to a surgical procedure performed during the hens’ 10th week of life. In terms of rearing efficiency, poulards had a more favorable feed conversion ratio (FCR) of 6.79 kg/kg, while that of hens was higher at 7.12 kg/kg, however, at P ≥ 0.05.

**Table 2:** Mortality and health cullings and use feed on 1 kgs of body weight.

Item	Mortality and health cullings					Feed conversion ratio FCR (kg/kg)
	number of birds				%	
	0 – 7 week	8 – 16 week	17 – 24 week	Total		
Hens (Group I)	-	-	-	0	0.00	7.12
Poulards (Group II)	-	1	-	1	2.50	6.79

Serum estradiol levels in the birds are shown in Fig. 1. At 10 weeks of age, no significant differences were observed between hens and poulards. However, at 12 and 24 weeks of age, poulards had significantly lower estradiol levels compared to hens (69.42 and 85.03 vs. 182.14 and 268.27 pg/ml), with statistically significant differences (P ≤ 0.01).

**Fig. 1:**
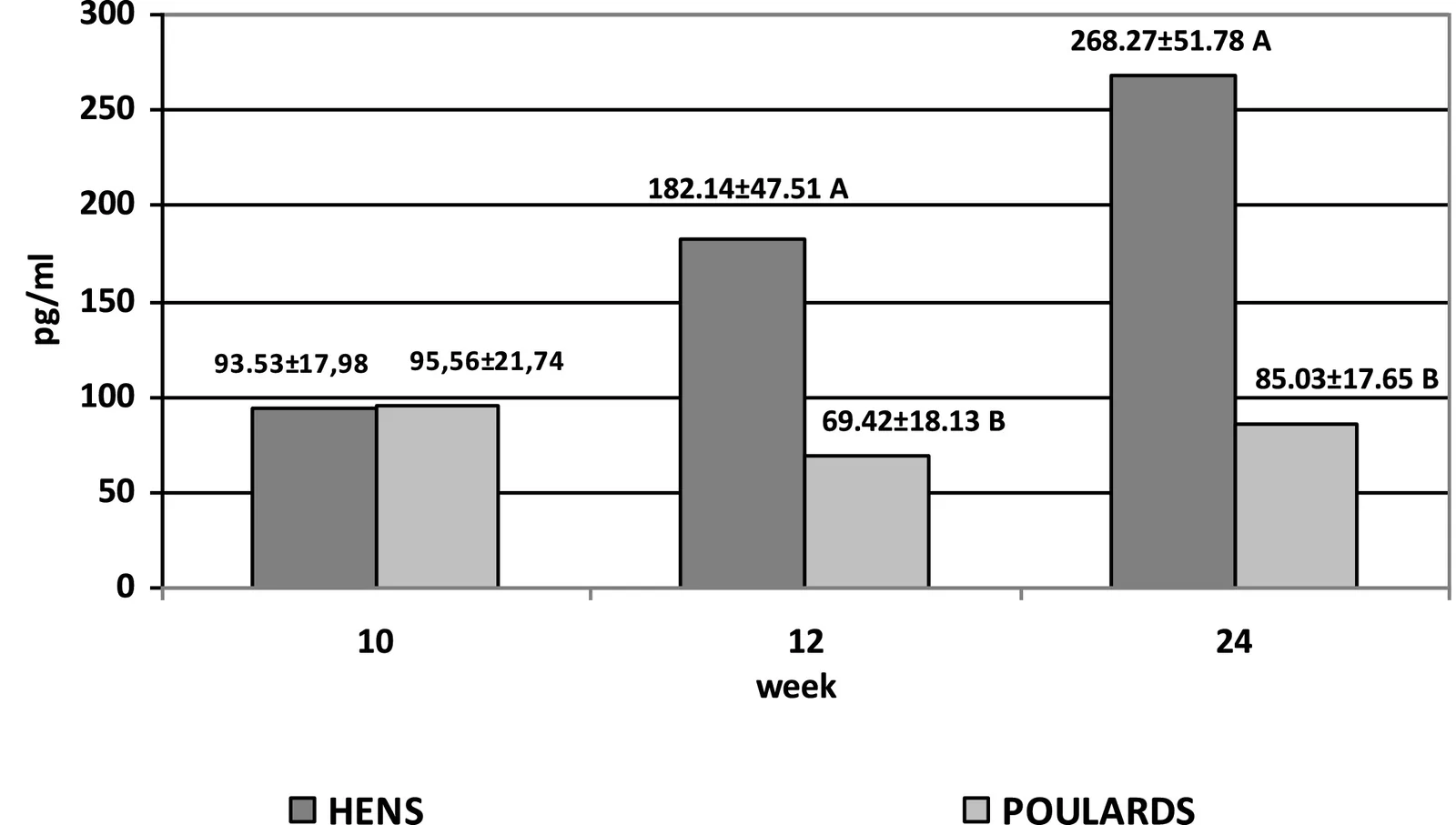
Blood estradiol levels in birds (pg/ml) of different ages.

Notes: A, B - as above for *P* ≤ 0.01.

The results of the biochemical blood analyses are presented in Table 3. In poulards, significantly lower values were observed for cholesterol (P ≤ 0.05) and triglycerides (P ≤ 0.01), while higher values were observed for amylase activity. No significant differences were found for ALT, AST, UREA, CREA, or LIPA.

**Table 3:** The blood indices of birds.

Item	Hens (Group I) x±SD	Poulards (Group II) x±SD
Chol (mg/dl)	122.20^a^ ± 12.64	102.04^b^ ± 9.82
ALT (u/L)	28.00 ± 8.03	26.80 ± 2.59
AST (u/L)	240.80 ± 37.62	219.00 ± 24.94
UREA (mg/dL)	14.40 ± 6.28	15.70 ± 1.83
Crea (mg/dL)	0.28 ± 0.07	0.29 ± 0.02
TG (mg/dL)	1139.00^A^ ± 336.66	48.20^B^ ± 12.85
AMYL (u/L)	326.40^B^ ± 47.69	703.20^A^ ± 72.94
LIPA (u/L)	18.16 ± 8.09	15.36 ± 1.67

Notes: x-mean value; SD-standard deviation;.

Chol-cholesterol; ALT-alanine transaminase; AST-aspartate transaminase; UREA-urea acid; Crea-enzymatic creatinine; TG-trigliceroles; AMYL-amylase; LIPA-lipase;.

a, b - values in rows with different letters differ significantly *P* ≤ 0.05.

A, B - as above for *P* ≤ 0.01.

As shown in Table 4, poulards (group II) had a significantly (P ≤ 0.05) higher body weight (by 63 g) at 24 weeks of age than hens (group I). In the group of spayed birds (group II), there was also a tendency toward higher carcass yield, both with and without giblets. Furthermore, poulards were distinguished by a significantly (P ≤ 0.01) higher proportion of breast muscle (18.72 % vs. 16.38 %) and leg muscle (23.03 % vs. 19.35 %). They also exhibited significantly (P ≤ 0.05 or P ≤ 0.01) higher liver weight (by 0.27 percentage points), abdominal fat thickness (by 1.32 percentage points), and leg bone percentage (by 0.37 percentage points). The poulard carcasses were also characterized by significantly (P ≤ 0.05 or P ≤ 0.01) higher values of the L* parameter (lighter color) and b* parameter (yellowness), as well as lower values of the a* parameter (redness).

**Table 4:** Body weight and results of slaughter analysis.

Item	Hens (Group I) x±SD	Poulards (Group II) x±SD
Live body weight (g)	1600^b^ ± 44.16	1663^a^ ± 41.67
Dressing percentage with giblets (%)	71.10 ± 1.73	72.87 ± 1.03
Dressing percentage without giblets (%)	66.87 ± 1.99	69.24 ± 1.90
Content in carcass:		
Breast muscles (%)	16.38^B^ ± 0.82	18.72^A^ ± 1.11
Leg muscles (%)	19.35^B^ ± 1.13	23.03^A^ ± 0.67
Giblets (%)	5.24 ± 0.47	5.45 ± 0.32
Liver (%)	1.90^b^ ± 0.17	2.17^a^ ± 0.17
Gizzard (%)	2.71 ± 0.35	2.71 ± 0.23
Heart (%)	0.63 ± 0.11	0.56 ± 0.06
Leg bones (%)	4.66^B^ ± 0.24	5.03^A^ ± 0.47
Abdominal fat (%)	2.39^b^ ± 0.57	3.71^a^ ± 0.66
Carcass color:		
– L*	68.21^b^ ± 1.10	70.36^a^ ± 1.18
– a*	4.29^A^ ± 0.22	3.62^B^ ± 0.48
– b*	11.37^B^ ± 2.14	15.74^A^ ± 2.52

Notes: x-mean value; SD-standard deviation;.

a, b - values in rows with different letters differ significantly *P* ≤ 0.05.

A, B - as above for *P* ≤ 0.01.

The physical parameters of the muscle are presented in Table 5. No statistically significant differences were found in pH values measured 15 min and 24 hours after slaughter in either the breast or leg muscles. In contrast, the breast muscles of poulards were characterized by significantly (P ≤ 0.01) lower drip loss and a tendency toward lower cooking losses. Similar relationships were observed in the leg muscles, although they were statistically significant (P ≤ 0.05) only for cooking losses. When color was assessed, it was found that the breast and leg muscles of poulards (group II), in contrast to those of hens, were significantly (P ≤ 0.05 or P ≤ 0.01) lighter (L*), more yellow, and less red. The breast and leg muscles of poulards (group II) also exhibited significantly (P ≤ 0.01) lower shear force, indicating greater tenderness. Furthermore, the evaluated muscle parts were characterized by significantly higher water holding capacity, which was statistically confirmed (P ≤ 0.01).

**Table 5:** Technological parameters of breast and leg muscles.

Item	Breast muscles		Leg muscles	
	Hens (Group I) x±SD	Poulards (Group II) x±SD	Hens (Group I) x±SD	Poulards (Group II) x±SD
pH_15_	6.13 ± 0.16	6.10 ± 0.20	6.38 ± 0.14	6.42 ± 0.08
pH_24_	5.92 ± 0.07	5.90 ± 0.07	6.11 ± 0.07	6.17 ± 0.08
Drip loss after 24 h (%)	0.69^B^ ± 0.11	0.46^A^ ± 0.07	0.44 ± 0.08	0.37 ± 0.05
Thermal loss (%)	23.79 ± 2.72	21.27 ± 3.59	34.96^a^ ± 2.83	32.24^b^ ± 1.86
Color:				
- L*	54.31^B^ ± 1.05	57.03^A^ ± 1.61	44.50^B^ ± 1.92	47.84^A^ ± 1.26
- a*	14.62^A^ ± 0.68	12.51^B^ ± 1.84	18.91^a^ ± 0.63	16.93^b^ ± 0.98
- b*	9.14^B^ ± 0.52	11.97^A^ ± 1.45	7.46^B^ ± 0.62	9.09^A^ ± 0.89
Tenderness (N)	18.25^A^ ± 1.09	13.71^B^ ± 0.84	25.16^A^ ± 1.64	20.88^B^ ± 1.60
Water holding capacity (%)	17.56^A^ ± 1.26	14.74^B^ ± 0.72	18.53^A^ ± 2.34	15.46^B^ ± 1.20

Notes: x-mean value; SD-standard deviation;.

a, b - values in rows with different letters differ significantly *P* ≤ 0.05.

A, B - as above for *P* ≤ 0.01.

As shown in Table 6, the chemical composition of the breast and leg muscles was similar in both groups evaluated. In the breast muscles of poulards (group II), only significant (P ≤ 0.05) differences in collagen content were observed. In contrast, in the leg muscles of poulards, significantly (P ≤ 0.05) higher levels of both ash and cholesterol were recorded. The results regarding fatty acid content in breast and leg muscles are presented in Table 7. Analysis of the fatty acid profile in breast muscles revealed no significant differences in most of the parameters examined, including the total content of saturated fatty acids (SFA). The dominant fatty acid in both groups was oleic acid (C 18:1). A statistically significant difference was observed only for the sum of n-3 polyunsaturated fatty acids (∑ PUFA n-3), which was higher in the breast muscles of poulards (by 0.22 percentage points). The docosahexaenoic acid (DHA) content was at a similar level, and the n-6 to n-3 fatty acid ratio was also similar in both groups, hovering around 9.5. More statistically significant differences were observed in the leg muscles, particularly regarding monounsaturated fatty acids. Compared to poulards, hens had a significantly (P ≤ 0.05) higher (by 3.23 percentage points) proportion of oleic acid (C₁₈:₁), which translated into a significantly (P ≤ 0.05) higher (by 4.25 percentage points) total MUFA content. The total content of saturated fatty acids (SFA) in the muscle was comparable in both groups and amounted to 34.91 g/100 g in hens and 35.95 g/100 g in poulards, respectively. Although a higher (by 3.22 percentage points) value for the sum of polyunsaturated fatty acids (∑ PUFA) was observed in poulards, this difference was not statistically significant. As with the breast muscles, the n-6 to n-3 fatty acid ratio in the leg muscles was similar and showed no statistically significant differences. The study revealed no statistically significant differences in the PI, AI, and TI indices for either breast or leg muscles.

**Table 6:** Results of effect of sterilization on chemical analysis of the breast and leg muscles.

Item	Breast muscles		Leg muscles	
	Hens (Group I) x±SD	Poulards (Group II) x±SD	Hens (Group I) x±SD	Poulards (Group II) x±SD
Dry matter (%)	26.74 ± 0.25	26.86 ± 0.28	26.16 ± 0.66	25.99 ± 0.64
Crude ash (%)	1.14 ± 0.02	1.13 ± 0.02	1.05^b^ ±0.03	1.09^a^ ± 0.02
Crude protein (%)	25.09 ± 0.30	25.46 ± 0.35	20.47 ± 0.38	20.74 ± 0.43
Crude fat (%)	1.06 ± 0.20	1.05 ± 0.16	4.80 ± 0.74	4.46 ± 0.66
Colagen [g/100 g]	0.45^b^ ± 0.03	0.52^a^ ± 0.03	0.97 ± 0.06	1.11 ± 0.19
Cholesterol (mg/100 g)	0.57 ± 0.05	0.57 ± 0.06	0.92^b^ ± 0.14	1.00^a^ ± 0.05

Notes: x-mean value; SD-standard deviation;.

a, b - values in rows with different letters differ significantly *P* ≤ 0.05.

A, B - as above for *P* ≤ 0.01.

**Table 7:** Results of the fatty acid profile of breast and leg muscles (g/100 g).

Item	Breast muscles		Leg muscles	
	Hens (Group I) x±SD	Poulards (Group II) x±SD	Hens (Group I) x±SD	Poulards (Group II) x±SD
C _14:0_	0.60 ± 0.08	0.57± 0.09	0.78 ± 0.04	0.74 ± 0.09
C _16:0_	25.58 ± 0.75	25.37 ± 1.49	24.88 ± 1.17	25.17 ± 1.36
C _16:1_	3.56 ± 0.77	2.91 ± 0.75	5.49 ± 0.83	4.49 ± 1.14
C _18:0_	11.07 ± 0.70	11.63 ± 0.80	9.06 ± 0.70	9.84 ± 0.94
C _18:1_	39.77 ± 1.64	37.52 ± 1.56	45.62^a^ ± 1.38	42.39^b^ ± 1.74
C _18:2n-6_	8.52 ± 0.29	10.57 ± 1.53	9.79 ± 0.71	12.66 ± 2.41
C _18:3n-3_	0.09 ± 0.02	0.07 ± 0.02	0.69 ± 0.08	0.89 ± 0.09
C_22_	0.29 ± 0.05	0.30 ± 0.04	0.12 ± 0.02	0.13 ± 0.02
C _20:4n-6_	8.60 ± 2.14	8.90 ± 1.28	2.92 ± 0.34	3.07 ± 0.66
C _22:6n-3_(DHA)	1.34 ± 0.29	1.43 ± 0.20	0.38 ± 0.03	0.40 ± 0.08
∑ SFA	37.58 ± 0.69	37.93 ± 0.97	34.91 ± 1.58	35.95 ± 0.66
∑ UFA	62.42 ± 0.69	62.07 ± 0.97	65.09 ± 1.58	64.05 ± 0.66
∑ MUFA	43.38 ± 2.11	40.48 ± 2.23	51.15^a^ ± 1.45	46.90^b^ ± 2.69
∑ PUFA	19.04 ± 2.36	21.59 ± 2.95	13.93 ± 0.84	17.15 ± 3.11
∑ PUFA_n-6_	17.21 ± 2.15	19.55 ± 2.73	12.84 ± 0.83	15.83 ± 2.97
∑ PUFA_n-3_	1.82^B^ ± 0.23	2.04^A^± 0.25	1.09 ± 0.09	1.32 ± 0.15
PUFA_n-6/n-3_	9.46 ± 0.54	9.56 ± 0.64	11.80 ± 1.27	11.96 ± 1.18
PI	45,01±8,32	48,43±6,53	24,38±1,55	28,11±4,87
TI	0,82±0,03	0,82±0,04	0,74±0,06	0,76±0,04
AI	0,45±0,02	0,45±0,04	0,43±0,03	0,44±0,03

Notes: x-mean value; SD-standard deviation;.

a, b - values in rows with different letters differ significantly *P* ≤ 0.05.

A, B - as above for *P* ≤ 0.01.

Sensory evaluation of the breast and leg muscles (Table 8) revealed a clear advantage for poulards (group II) over hens (group I) in all evaluated traits. The breast muscles of poulards received significantly (P ≤ 0.01) higher scores for aroma (by 0.50 percentage points) and palatability (by 1.06 percentage points) Furthermore, the evaluated muscles were characterized by better juiciness (by 0.67 percentage points) and tenderness (by 0.56 percentage points), which was confirmed at the P ≤ 0.05 significance level. Even more pronounced differences were observed in the evaluation of leg muscles, where poulards (group II), compared to the hen group (group I), received significantly (P ≤ 0.01) higher scores for all evaluated parameters, namely: aroma, juiciness, tenderness, and palatability (4.72-4.77 points vs. 4.11-4.22 points).

**Table 8:** Results of sensory analysis of breast and leg muscles.

Item	Breast muscles		Leg muscles	
	Hens (Group I) x±SD	Poulards (Group II) x±SD	Hens (Group I) x±SD	Poulards (Group II) x±SD
Aroma (pts.)	4.27^B^ ± 0.26	4.77^A^ ± 0.36	4.22^B^ ± 0.66	4.77^A^ ± 0.36
Juiciness (pts.)	3.88^b^ ± 0.54	4.55^a^ ± 0.39	4.11^B^ ± 0.54	4.72^A^ ± 0.36
Tenderness (pts.)	4.16^b^ ± 0.55	4.72^a^ ± 0.44	4.11^B^ ± 0.48	4.72^A^ ± 0.36
Flavor (pts.)	3.66^B^ ± 0.79	4.72^A^ ± 0.44	4.16^B^ ± 0.43	4.72^A^ ± 0.36

Notes: x-mean value; SD-standard deviation;.

a, b - values in rows with different letters differ significantly *P* ≤ 0.05.

A, B - as above for *P* ≤ 0.01.

## Discussion

In the studies conducted, the low mortality rate in all groups and the absence of culling for health reasons indicate that the poulardization procedure used is highly safe. Similar results were obtained by Calik et al. (2020), Calik and Obrzut, 2023a. Rearing efficiency, expressed as the feed conversion ratio (FCR), was more favorable in poulards. Baeza et al. (2010) and Calik et al. (2017) demonstrated better feed utilization in castrated birds. It was observed that a decrease in male sex hormone levels is associated with lower motor activity in capons, which contributes to better feed utilization and greater weight gains. Similar relationships were described by Kwiecień et al. (2015, 2018), Murawska et al. (2019), and Sumon et al. (2024), pointing to improved rearing performance and feed conversion in sterilized birds.

As reported by Guo et al. (2017), ovariectomy – the surgical removal of the ovary – in hens results in a rapid decrease in blood estradiol levels. Significantly lower estradiol levels in poulards at 12 and 24 weeks of age, compared to hens, confirm the effectiveness of the ovariectomy procedure and the permanent nature of the changes in hormonal balance caused by ovary removal, which was also observed in earlier studies by Guo et al. (2017) and Obrzut et al. (2018). This phenomenon has been repeatedly described in the literature, where a link has been demonstrated between reduced levels of estrogens and androgens and increased muscle and fat tissue deposition in sterilized birds (Sirri et al., 2009; Gesek et al., 2017; Zawacka et al., 2017; Calik and Obrzut, 2023a).

In our own studies on poulards, we observed lower levels of cholesterol and triglycerides and higher amylase activity. Different results were obtained by Kasperek et al. (2021) for sterilized roosters in terms of cholesterol and triglyceride levels, but consistent results regarding amylase activity. The cited authors did not observe any statistically significant differences in ALT, AST, UREA, CREA, or LIPA levels, a finding also observed in our own studies. Cui et al. (2016b) demonstrated significantly higher triglyceride and total fat content in the liver of sterilized females and males. Zhang et al. (2025) found no significant differences between the control group (hens) and the experimental group (ovariectomized hens) regarding alanine aminotransferase (ALT) activity, triglyceride (TG) levels, and creatine kinase (CK) activity, indicating that liver function, lipid metabolism, and markers of muscle damage remained stable under the conditions of this study.

Our study showed that poulards, compared to hens, had higher body weight, greater proportions of breast and leg muscle, liver, and bone, as well as higher carcass fat content – a typical effect of reduced gonadal activity and decreased estradiol levels. These results are consistent with the studies by Berri et al. (2005), Guo et al. (2015, 2017), Kwiecień (2018), Kasperek et al. (2021) and Zawacka et al. (2017), who demonstrated that castration of native-breed hens and roosters leads to an increase in carcass weight, breast muscle percentage, and fat cover, while simultaneously improving slaughter value. Research conducted by Zhang et al. (2025) indicates that surgical ovariectomy in Wuding hens significantly affects growth, carcass traits, muscle quality, and the metabolic remodeling of skeletal muscle. The authors emphasize that, following the onset of laying, a large proportion of nutrients in hens is allocated to egg production, whereas in ovariectomized hens, it is directed toward somatic growth – a point also highlighted by Cui et al. (2016b). The results of studies by Mahmud et al. (2013) also indicate significantly higher liver weight in castrated roosters. A similar relationship was observed in our study, and the significant difference may stem from the fact that the liver is the primary site of fatty acid synthesis in birds.

The evaluated muscle parts from poulards (breasts and legs) exhibited lower drip and cooking losses, as well as a more favorable lower shear force, indicating better tenderness and juiciness. These findings are consistent with the results of studies by Berri et al. (2005), Cui et al. (2016a), Krawczyk et al. (2018), Calik and Obrzut (2023a), Kasperek et al. (2021). The cited authors indicated that a slower growth rate and modified hormonal metabolism in the birds (caponization) promote a more favorable muscle fiber structure and improved meat texture parameters. Improved water holding capacity in the leg muscles of poulards was also noted in our earlier studies (Obrzut et al., 2018) and in the studies of Zhang et al. (2025). Similar results from studies conducted on capons were also observed by Wojtysiak et al. (2019). The authors emphasize that this may be related to changes in muscle fiber structure and a higher proportion of intramuscular fat. From a technological standpoint, the combination of lower drip loss, better water holding capacity, and lower shear force is more advantageous, as also indicated by the results of studies by Diaz (2010) and Kasperek (2023). Our own studies showed that both the carcass color and the color of the breast and leg muscles of poulards were significantly lighter (L*), more yellow, and less red, which is consistent with the results of studies on capons (Sirri et al., 2009; Guo et al., 2015; Franco et al., 2016; Calik et al., 2017, 2020). Likewise, the study by Cui et al. (2016a) demonstrated similar relationships in the breast muscles of poulards. In contrast, regarding breast muscle color, Zhang et al. (2025) observed reduced lightness (L*) and increased redness (a*) in hens ovariectomized at 240 days of age compared to birds of other ages; this may have been associated with increased myoglobin concentration or the maturation of muscle fibers.

In poulards and hens, very similar dry matter, protein, and fat contents were found, with only slight differences in ash and collagen content in the leg muscles. While Sirri et al. (2009), Kwiecień et al. (2018), described significantly higher levels of total fat, protein, and ash in the muscles of castrated birds.

The results of Calik and Obrzut (2023a) also indicate a significantly higher collagen content in the breast muscles of capons. Similarly, Sokołowicz et al. (2016) report that the meat of castrated animals has a lower collagen content, which in turn results in better tenderness – a finding also observed in our own studies. In the study by Obrzut et al. (2018), poulards were found to have a significantly higher dry matter content in breast muscles, as well as higher dry matter and fat content in leg muscles. The minor changes in chemical composition observed in this study may result from differences in sex (females vs. males in most studies), age at slaughter, and the specific characteristics of conservation breeds, in which the rate of fat deposition and muscle development differs from that of intensively reared commercial lines. The fatty acid profile of breast and leg muscles in chickens and poulards was similar, with a predominant proportion of oleic acid and a moderate proportion of polyunsaturated fatty acids, with only a slight increase in the proportion of PUFAs and n-3 fatty acids in the leg muscles of poulards. In our previous studies as well, the differences were minor and generally not significant (Obrzut et al., 2018). In contrast, the literature on native-breed capons has described a higher proportion of PUFAs, including n-3, as well as a favorable PUFA/SFA and n-6/n-3 ratios in the muscles of birds that underwent the procedure (Kwiecień et al., 2018; Calik and Obrzut, 2023a). The AI and TI values obtained for the hens' muscles were lower than those reported in studies by Popova et al. (2016) and Sosnówka-Czajka et al. (2017). As noted by Dal Bosco et al. (2022), lower AI and TI values suggest a protective potential for coronary artery health; therefore, it is recommended that these values remain below 1.0 and 0.5, respectively. In our study, neither the hens nor the poulards exceeded these thresholds. However, the estimated PI – an indicator of fat susceptibility to oxidation – was higher in the breast muscles and lower in the leg muscles compared to the results reported by Kasperek et al. (2023), who found statistically significant differences between roosters and capons regarding leg muscles.

Higher sensory ratings for poulard meat – particularly in terms of tenderness, juiciness, and flavor – confirm that ovariectomy improves the product’s consumer qualities. The improved tenderness and increased juiciness of poulard meat are associated with a higher fat content and a favorable fatty acid profile, as highlighted by Diaz (2010), Baeza et al. (2010), Gesek (2017, 2019), Wojtysiak et al. (2019) and Calik et al. (2020). According to Calik et al. (2017), intramuscular fat acts as a carrier of aromatic compounds, and its presence increases the perceived juiciness and palatability of the meat, which is reflected in higher sensory ratings of meat from castrated birds compared to uncastrated ones. From a practical standpoint, the obtained results align with the concept of using conservation breeds for the production of niche meat with enhanced sensory quality, discussed, among others, in the review by Sokołowicz et al. (2016), which highlights the potential of native breeds as source material for the production of capons and poulards. This is confirmed by studies by Duran (2004); Calik et al. (2015, 2020), and Calik and Obrzut (2025) on capons of local breeds, which demonstrated that the meat of such birds is distinguished by characteristics sought after by a growing group of health-conscious consumers – better flavor, tenderness, and distinctive color – a finding that was also fully confirmed in the present study. It can therefore be concluded that the ovariectomy of hens from conservation breeds to produce poulards is a valuable tool for creating a premium product that combines the conservation of genetic resources with the development of a high-quality market offering.

In conclusion, based on the results obtained, it was found that, poulards achieve higher body weight and slaughter yield. In addition, they are characterized by a higher proportion of breast and leg muscles in the carcass, as well as higher fat content. The color of poulard carcasses and meat was lighter and more yellow, and less red (higher L* and b*, lower a*), which is consistent with the typical appearance of meat from castrated birds and may be viewed as a desirable trait in the premium product segment. Compared to hen meat, poulard meat exhibited more favorable physical properties, i.e., lower shear force, lower drip loss after 24 hours, and lower cooking losses – which indicates its superior technological quality. Poulard meat was rated higher than hen meat in terms of aroma, juiciness, tenderness, and flavor, indicating its superior culinary qualities. The basic chemical composition of the meat (dry matter, protein, fat) and the overall SFA/UFA ratios were similar in hens and poulards; only slight differences were observed in ash content, collagen, and the proportion of PUFAs in leg muscles, favoring the spayed birds. The study results confirm that hens of conservation breeds are a valuable source for poulard meat production – combining better sensory quality and distinctive product characteristics with the goal of biodiversity conservation. This is consistent with the concept of using native breeds to produce high-quality niche culinary products.

## Author contributions

**Joanna Obrzut** was responsible for conceiving and designing the study, with designing the methodology, conducting a literature search, extracting and analysing the data, interpreting the findings, writing the manuscript and coordinating the review process.

**Jolanta Calik** helped with designing the study, with the design of the methodological approach, conducting the literature search, extracting the data, interpreting the findings and critically reviewing the manuscript. All authors made substantial intellectual contributions to the conception, design, analysis and interpretation of the work. They were involved in drafting and critically revising the manuscript for its intellectual content, and reviewed and approved the final version submitted for publication. They are accountable for all aspects of the work and will ensure that any questions regarding the accuracy or integrity of any part of the article are appropriately addressed.

## Disclosures

The authors declare that they have no known competing financial interests or personal relationships that could have appeared to influence the work reported in this paper.
